# Investigating Safety and Technological Traits of a Leading Probiotic Species: *Lacticaseibacillus paracasei*

**DOI:** 10.3390/nu16142212

**Published:** 2024-07-10

**Authors:** Andrea Colautti, Federica Ginaldi, Lucia Camprini, Giuseppe Comi, Anna Reale, Lucilla Iacumin

**Affiliations:** 1 Department of Agrifood, Environmental and Animal Science, University of Udine, Via Sondrio 2/A, 33100 Udine, Italygiuseppe.comi@uniud.it (G.C.); 2 Institute of Food Science (ISA), National Research Council, Via Roma, 64, 83100 Avellino, Italy; anna.reale@isa.cnr.it

**Keywords:** probiotics, fermentations, food safety, food technology, *Lacticaseibacillus* spp., lactic acid bacteria

## Abstract

*Lacticaseibacillus* spp. are genetically close lactic acid bacteria species widely used in fermented products for their technological properties as well as their proven beneficial effects on human and animal health. This study, the first to include such a large collection of heterogeneous isolates (121) obtained from international collections belonging to *Lacticaseibacillus paracasei*, aimed to characterize the safety traits and technological properties of this important probiotic species, also making comparisons with other genetically related species, such as *Lacticaseibacillus casei* and *Lacticaseibacillus zeae*. These strains were isolated from a variety of heterogeneous sources, including dairy products, sourdoughs, wine, must, and human body excreta. After a preliminary molecular characterization using repetitive element palindromic PCR (Rep-PCR), Random Amplification of Polymorphic DNA (RAPD), and Sau-PCR, particular attention was paid to safety traits, evaluating antibiotic resistance profiles, biogenic amine (BA) production, the presence of genes related to the production of ethyl carbamate and diaminobenzidine (DAB), and multicopper oxidase activity (MCO). The technological characteristics of the strains, such as the capability to grow at different NaCl and ethanol concentrations and different pH values, were also investigated, as well as the production of bacteriocins. From the obtained results, it was observed that strains isolated from the same type of matrix often shared similar genetic characteristics. However, phenotypic traits were strain-specific. This underscored the vast potential of the different strains to be used for various purposes, from probiotics to bioprotective and starter cultures for food and feed production, highlighting the importance of conducting comprehensive evaluations to identify the most suitable strain for each purpose with the final aim of promoting human health.

## 1. Introduction

The revised *Lactobacillus* genus, recently reclassified into 25 genera, comprises 261 bacterial species, including many renowned for their technological traits [1,2]. Among these, the newly categorized *Lacticaseibacillus* genus is particularly noteworthy, containing two genetically correlated species of significant interest—*Lacticaseibacillus paracasei* and *Lacticaseibacillus casei*—from which *Lacticaseibacillus zeae* was further differentiated [3]. These closely related species were formerly challenging to distinguish taxonomically before the widespread use of Next-Generation Sequencing (NGS) techniques [4]. They are found in diverse environments such as the gastrointestinal tract of animals and humans, as well as various foods and beverages like milk, meat, vegetables, cereals, beer, and wine, and have garnered significant interest [5]. The importance of *Lbs. paracasei* is particularly notable for its role in modulating the gut microbiota, which is now understood to be far more complex and influential than previously thought [6,7]. Numerous strains of *Lbs. casei*, *Lbs. zeae*, and *Lbs. paracasei* find application in food preservation and serve as starters for fermented dairy, plant-based, and meat products. Furthermore, an expanding range of commercial products leverage the probiotic properties of these lactic acid bacteria, either alone or incorporated into foods, to promote human health by modulating gastrointestinal microbiota and producing beneficial metabolites [8]. 

Given their increasing relevance, it becomes imperative to assess specific properties of new strains to enhance understanding and explore new applications. The study of *Lbs. paracasei* opens new avenues in the development of both traditional and innovative probiotics, prebiotics, and postbiotics, enhancing their effectiveness and expanding their use. This includes exploring how *Lbs. paracasei* can contribute to broader health benefits beyond gut health, such as influencing the gut–brain axis; managing chronic inflammation; and addressing autoimmune disorders, arthritis, obesity, skin disorders, and food allergies. In this regard, it is crucial that these strains withstand the stresses of industrial processes and adapt to the physicochemical characteristics of the matrix into which they are inoculated.

Despite their generally recognized as safe (GRAS) status, all strains used in the food sector must undergo taxonomic identification and genetic and phenotypic characterization, as recommended by EFSA [9,10,11]. Beyond identifying resistance and virulence factors that may pose health risks to vulnerable individuals or genes transferable via horizontal transmission to other microorganisms [12,13], it is essential to investigate the presence of harmful metabolisms such as biogenic amines (BAs) and ethyl carbamate production. BAs, primarily produced by the microbial decarboxylation of amino acids found in many foods, can have toxicological effects if ingested in large amounts, causing allergic reactions, vomiting, hypertension, and headaches [14,15]. However, certain *Lacticaseibacillus* spp. strains possess enzymes capable of degrading these compounds, offering a potential solution [16].

Therefore, this study aimed to investigate the genetic heterogeneity of a large collection consisting of 121 *Lbs. paracasei* strains, which were also compared with 6 *Lbs. casei* and 2 *Lbs. zeae* strains. The smaller number of *Lbs. casei* and *Lbs. zeae* strains in this study is due to the difficulty of isolating them in nature and obtaining them from collections. However, considering their close genetic correlation, their inclusion in the characterization allowed for better defining the borders among the species and aimed to shed light on possible species-specific traits. The microorganisms, previously identified [4] using species-specific PCRs and high-resolution melting analysis (HRM), underwent genetic characterization via Rep-PCR, RAPD, and Sau-PCR. The evaluation also encompassed assessing the presence of *arcABC* genes related to ethyl carbamate metabolism and BA production, as well as their growth capabilities under various conditions, including different concentrations of NaCl and ethanol, pH levels, antibiotic resistance, production of BAs, BA-degrading activity, and production of antimicrobial compounds. This comprehensive assessment, conducted across a significant number of strains, aimed to broaden our understanding of both the genetic and phenotypic characteristics of these three species, which are of both technological and food safety importance.

## 2. Materials and Methods

### 2.1. Bacterial Strains and Culture Conditions

The strains, originating from various sources or obtained from international collections, were gathered by the University of Udine, University of Basilicata, and University of Molise, resulting in a total of 129 strains belonging to the species *Lbs. casei* (6 strains), *Lbs. zeae* (2 strains), and *Lbs. paracasei* (121 strains) (Table 1). Their identification was previously conducted, as documented in a prior study, through species-specific PCR followed by HRM (high-resolution melting) analysis [4]. The strains were cryopreserved at −80 °C in cryovials containing DeMan, Rogosa, and Sharpe broth (MRS, Oxoid, Waltham, MA, USA) supplemented with 20% glycerol. Prior to subsequent analyses, they were streaked twice on MRS Agar and incubated under microaerophilic conditions at 30 °C for 48 h to check their purity.

### 2.2. DNA Extraction from Pure Colonies

For DNA extraction, pure colonies were cultured for 48 h at 30 °C in MRS broth (Oxoid). Subsequently, 2 mL of the cultures was centrifuged at 10,000× *g* for 10 min at 4 °C to pellet and harvest the cells. The DNA extraction was performed using the MasterPureComplete™ DNA & RNA Purification Kit (Epicentre Biotechnologies, Madison, USA) following the manufacturer’s instructions.

### 2.3. Rep-PCR, RAPD, and Sau-PCR Analyses

The molecular characterization was performed according to Iacumin et al. 2020 [17]. A Rep-PCR analysis was performed using the (GTG)_5_ primer (5′-GTGGTGGTGGTGGTG-3′) [18], using the following reaction mix: 10 mM Tris-HCl (pH 8.3), 50 mM KCl, 1.5 mM MgCl_2_, 0.2 mM each dNTPs, 1 µM primer (GTG)_5_, 1.25 U/L Taq-polymerase (Applied Biosystem, Waltham, USA), and 100 ng of DNA, for a total volume of 25 µL. The reactions were carried out using a Euroclone Thermal Cycler (Celbio, Milan, Italy), and the amplification protocol consisted of 31 cycles of denaturation at 94 °C for 3 s, followed by one step at 92 °C for 30 s, annealing at 40 °C for 1 min, and extension at 65 °C for 8 min. The initial denaturation was at 95 °C for 2 min and the final extension at 65 °C for 8 min.

The RAPD analysis was performed using the M13 primer (5′-GAGGGTGGCGGTTCT-3′) [19], using the following reaction mix: 10 mM Tris-HCl (pH 8.3), 50 mM KCl, 1.5 mM MgCl_2_, 0.2 mM each dNTPs, 1 µM primer M13, 1.25 U/L Taq-polymerase (Applied Biosystem), and 100 ng of the extracted DNA, for a total volume of 25 µL. The reactions were carried out in a Euroclone Thermal Cycler (Celbio) and the amplification protocol consisted of 35 repetitions of 94 °C for 1 min, 38 °C for 1 min, ramp to 72 °C at 0.6 °C/s, and 72 °C for 2 min. At the beginning of the reaction, an initial denaturation step at 94 °C for 5 min was performed and a final extension at 72 °C for 5 min was carried out.

The Sau-PCR analysis was performed using one microliter of Sau3AI restriction endonuclease (10 U/μL) to digest 200 ng of DNA overnight at 37 °C in a final volume of 20 μL. After the enzymatic restriction, the amplification was performed using SAG1 primer (5′-CCGCCGCGATCAG-3′) [20], using the following reaction mix: 10 mM Tris–HCl (pH 8.3), 50 mM KCl, 1.5 mM MgCl_2_, 0.2 mM dNTPs, 2 µM primer SAG1, 1.25 U/L Taq-polymerase (Applied Biosystem), and 1 µL of the digested DNA, for a total volume of 50 µL. The reactions were carried out using an Euroclone Thermal Cycler (Celbio), using the following protocol: 25 °C for 5 min, ramp to 60 °C at 0.1 °C/s, 60 °C for 30 s, 2 cycles of 95 °C for 1 min, 50 °C for 15 s, ramp to 25 °C at 0.1 °C/s, ramp to 50 °C at 0.1 °C/s, 50 °C for 30 s, 35 cycles of 94 °C for 15 s, 46 °C for 1 min, 65 °C for 2 min, and a final extension at 65 °C for 2 min.

The PCR products obtained from these techniques were separated in a 1.5% (*w*/*v*) agarose gel in TBE 0.5× at 120 V, for 6 h for RAPD and Rep-PCR and 4 h for Sau-PCR, and stained for 30 min at the end of the electrophoretic run in TBE 0.5× buffer containing ethidium bromide 0.25 µL/mL (Merck, Darmstadt, Germany). The digital images of the gels were acquired through the BioImaging System GeneGenius imaging software v1.5.7.0 (Syngene, Bangalore, India).

The fingerprints analysis was carried out with the Gel Compare II software Version 4.1 (Applied Maths, Sint-Martens-Latem, Belgium), calculating their similarity using Pearson product-moment correlation coefficients. Dendrograms were obtained by means of the unweighted pair group method using arithmetic average (UPGMA) clustering algorithms [21].

### 2.4. Growth Capabilities

The resistance of the strains to stressful conditions was assessed by cultivating them in modified MRS broth. Initially, pure colonies were inoculated into MRS broth and incubated at 30 °C for 24 h. Subsequently, the cultures were centrifuged at 1500× *g* for 5 min, and the resulting pellets were washed twice with a maximum recovery diluent (0.1% *w*/*v* bacteriological peptone and 0.85% *w*/*v* NaCl at pH 7). The cellular pellets were then used to prepare standardized suspensions with an OD_600_ of 0.1 (corresponding to 10^7^ cfu/mL), which were inoculated in triplicate for each experiment in the modified media at a final concentration of 10^2^ CFU/mL. The analysis was conducted in the wells of a 96-well microplate with a U-bottom, containing a final volume of 200 μL of MRS broth modified with varying concentrations of NaCl (2%, 4%, and 6.5% *w*/*v*) or ethanol (12% and 15% *v*/*v*) or adjusted to different pH levels using HCl (pH 3.2, 3.8, 4.2, and 4.6). The microplates were then incubated at 30 °C, and the optical density at 600 nm was measured after 3, 24, and 48 h using a TECAN SUNRISE microplate reader (Tecan, Switzerland). Based on the change in optical density after 48 h of incubation, the strains were classified as sensitive (OD_600_ from 0 to 0.3), intermediate (OD_600_ from 0.3 to 0.9), or resistant (OD_600_ from 0.9 to 1.5) to the tested growth conditions.

### 2.5. Antibiotic Resistance

The antibiotic resistance profiles of the strains were determined using an antibiotic-susceptible disk diffusion assay (Oxoid) [22]. A panel of antibiotics was employed, including cefoperazone 30 µg (CFP30), cefazolin 30 µg (KZ30), chloramphenicol 10 µg (C10), clindamycin 10 µg (DA10), erythromycin 30 µg (E30), kanamycin 30 µg (K30), ofloxacin 5 µg (OFX5), quinupristin/dalfopristin 15 µg (QD15), rifampicin 30 µg (RD30), streptomycin 25 µg (S25), tetracycline 10 µg (TE10), tobramycin 10 µg (TOB10), and vancomycin 30 µg (VA30). For each trial, bacterial suspensions with an OD_600_ of 0.1 were prepared from a single colony inoculated in MRS broth and then incubated for 24 h at 37 °C. Subsequently, the antibiotic-susceptible disk diffusion assay was conducted on MRS agar plates that had been previously inoculated with 100 µL of the bacterial suspension. The plates were then incubated under microaerophilic conditions for 24 h at 37 °C. Following incubation, the diameters of the inhibition zones were measured using calipers, and the results were interpreted according to the method outlined by Charteris et al., 1998 [23], classifying each strain as sensitive (S), intermediate (I), or resistant (R) to each tested antibiotic. *Staphylococcus aureus* ATCC25923, *Escherichia coli* ATCC35218, *Pseudomonas aeruginosa* ATCC 27853, *Haemophilus influenza* ATCC49247, *Neisseria gonorrhoeae* ATCC49226, *Streptococcus pneumoniae* ATCC 49619, and *Escherichia coli* ATCC35218 were used as reference strains.

### 2.6. Antimicrobial Capabilities (Bacteriocin Production)

The production of antimicrobial peptides was assessed using an agar well diffusion assay [24]. To obtain the cell-free supernatant, cultures incubated overnight in MRS broth at 30 °C were centrifuged for 10 min at 7000× *g*. Subsequently, the supernatant was filtered through a 0.2 µm pore-size cellulose acetate filter. The filtered supernatant was then divided into three aliquots: one aliquot was used without any modification, one aliquot was pH-adjusted to 6.5 as a control to avoid the antimicrobial effect caused by acidity, and the last aliquot was treated with proteinase K (2 mg/mL, Merck) to deactivate any peptides with potential antimicrobial activity and catalase (1 mg/mL, Merck) to remove the hydrogen peroxide. The effects of these supernatants were tested on Brain Heart Infusion (Oxoid) soft agar plates (0.01 g/mL agar) inoculated with *Listeria monocytogenes* ATCC7644, *Staphylococcus aureus* DSM 4910, *Escherichia coli* DSA 21, or *Salmonella enteritidis* DSA 11 sourced from the strain collection of the University of Udine. Four wells, each 5 mm in diameter, were made in each plate and filled with 100 µL of the overnight LAB cultures, the filtered supernatant, the pH 6.5 filtered supernatant, or the pK-treated supernatant. Following incubation of the plates for 24 h at 37 °C, the presence of inhibition zones around the wells was observed to determine the antimicrobial activity. Trials were conducted in triplicate.

### 2.7. Biogenic Amine (BA) Production

Biogenic amine production was evaluated following the method described by Bover-Cid and Holzapfel, 1999 [25]. Briefly, a single bacterial colony was selected and inoculated into MRS broth supplemented with 0.1% of each amino acid precursor (tyrosine free base, histidine monohydrochloride, ornithine monohydrochloride, arginine hydrochloride, and lysine hydrochloride; Merck), along with 0.005% pyridoxal-5-phosphate (Merck), and then incubated overnight at 30 °C. This process was repeated five times, after which the cultures were transferred to Bover-Cid agar and broth mediums and incubated for 48 h at 30 °C. A color change from yellow to purple in both agar and broth indicated a positive result for specific biogenic amine production. Each trial was repeated in triplicate. Additionally, a multiplex PCR assay was performed to detect the presence of *hdc*, *tyrdc*, *agdi,* and *odc* genes, associated with the production of histamine, tyramine, ornithine, and putrescine, respectively, confirming the target gene by sequencing the PCR amplification products (Eurofins Genomics, Germany) according to the protocols described by Coton et al., 2010 [26]. *Enterococcus faecalis* EF37 (for *tyrdc* and *agdi*), *Streptococcus thermophilus* PRI60 (for *hdc*), and *Lacticaseibacillus rhamnosus* N132 (for *odc*) sourced from the collection of the University of Udine were used as positive controls.

### 2.8. arcABC Gene Presence

The strains were screened for the presence of *arcABC* genes, coding for enzymes involved in the arginine dihydrolase system (ADI) pathway in *Lacticaseibacillus* spp. To detect the presence of *arcABC* genes, degenerate primers addressing *arcA* (arginine deiminase), *arcB* (ornithine transcarbamylase), and *arcC* (carbamate kinase) genes that produced amplicons of 266 bp, 181 bp, and 343 bp, respectively, were used as described by Araque et al., 2009 [27]. *Lactiplantibacillus plantarum* 64 and *Lpb. plantarum* 70 (strain collection of the Viticulture and Oenology Department, Stellenbosch University, South Africa) were used as positive controls. After PCR, amplicons were resolved by electrophoresis on 1.5% (*w*/*v*) agarose gels in 0.5× TBE buffer supplemented with 0.025% (*v*/*v*) ethidium bromide (EtBr) and sequenced to confirm the target gene (Eurofins Genomics).

### 2.9. Diaminobenzidine (DAB) Assay and Multicopper Oxidase (MCO) Detection

The diaminobenzidine (DAB) assay and multicopper oxidase (MCO) detection were conducted according to the protocols outlined by Callejón et al., 2014 [28]. Initially, a single colony was inoculated onto modified MRS agar supplemented with L-cysteine (0.5 g/L) and biogenic amines (putrescine, tyramine, and histamine) at concentrations of 10 mg/L each, followed by overnight incubation at 37 °C. Subsequently, the overnight cultures underwent centrifugation at 10,000× *g* for 10 min, and the resulting cell pellets were washed twice with 25 mL of a 50 mM solution of sodium phosphate buffer (pH 7.4). After the second wash, the cells were resuspended in 500 µL of the same buffer supplemented with 1 mM phenylmethylsulfonyl fluoride (PMSF). The suspensions were transferred to 1.5 mL tubes containing 1 g of glass beads and subjected to agitation using a Qiagen Shaker for 10 cycles of 40 s each, with the tubes being placed on ice for 5 min after 5 cycles. To recover the cell extract, the tubes were then centrifuged at 10,000× *g* for 15 min. After, 25 µL of each sample was mixed with gel loading buffer 2× (Merck), loaded in a polyacrylamide gel (stacking 4% and non-denaturing 8%), and electrophoresed with a Miniprotean (BioRad, Hercules, CA, USA) for 1 h at 30 mA in Tris–glycine buffer (25 mM Tris base and 192 mM glycine). After the electrophoretic run, the gel was used to assess DAB and MCO activities. Amine-degrading activity was detected by placing the gel in a sodium phosphate buffer (50 mM, pH 7.4) containing 1 mM histamine, tyramine, and putrescine. After 15 min, the gel was transferred to a sodium phosphate buffer (50 mM, pH 7.4) supplemented with horseradish peroxidase (1000 U/L) and diaminobenzidine (DAB, 0.25 mM). After 3 h, the gels were examined: a brown color on the active band meant the presence of amine-degrading activity. MCO activity was assessed by immersing the gels in a sodium acetate buffer (100 mM, pH 4) containing 10 mM 2,6-dimethoxyphenol (DMP). After 5 min, the gels were transferred to the same buffer supplemented with 1 mM CuSO4. After 10 min, the gels were observed, and the presence of an orange-yellow band indicated MCO activity. Trials were conducted in triplicate for each strain.

## 3. Results and Discussion

### 3.1. Rep-PCR, RAPD, and Sau-PCR Molecular Characterization

**Figure 1:**
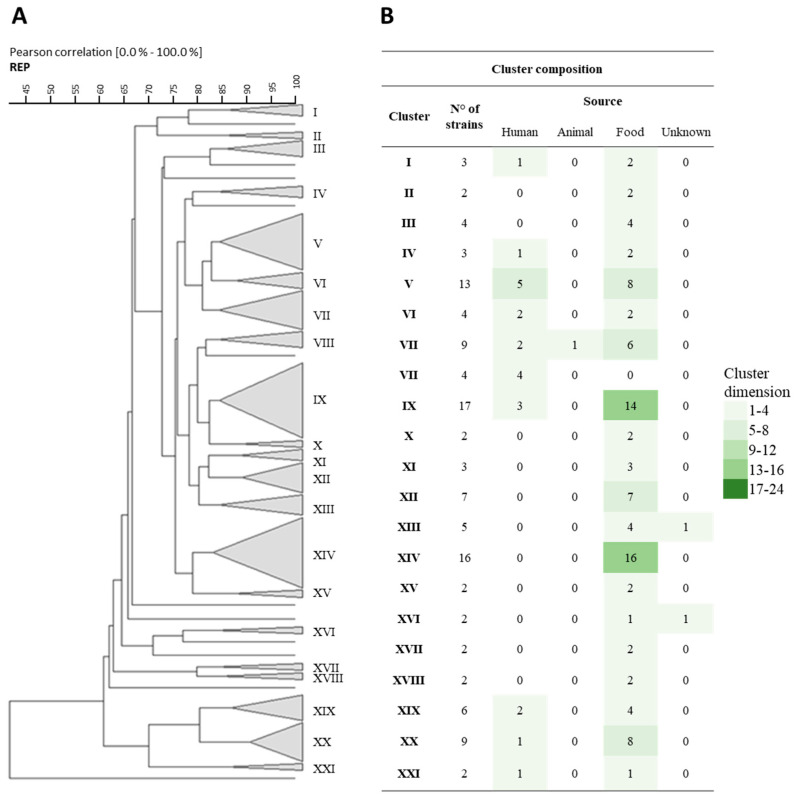
(**A**) Cluster analysis using Pearson product-moment correlation coefficients and unweighted pair group method using an arithmetic average (UPGMA) of the profiles obtained by Rep-PCR analysis of the different tested strains. A similarity coefficient of 83% was arbitrarily chosen. The identified clusters are indicated with Roman numerals. Strain DBPZ0420 did not provide a comparable Rep profile to the other strains, so it was not included in this figure. (**B**) The table within the figure shows the composition of the clusters, also in relation to their source of isolation.

The fingerprinting analysis revealed the formation of 21 clusters based on the Rep-PCR results, with an 83% similarity coefficient (Figure 1A). Despite the absence of correlations linked to species or geographical origin, three main clusters (V; IX; and, notably, cluster XIV, consisting solely of strains isolated from foods) displayed a notable correlation based on their isolation matrix (Figure 1B).

**Figure 2:**
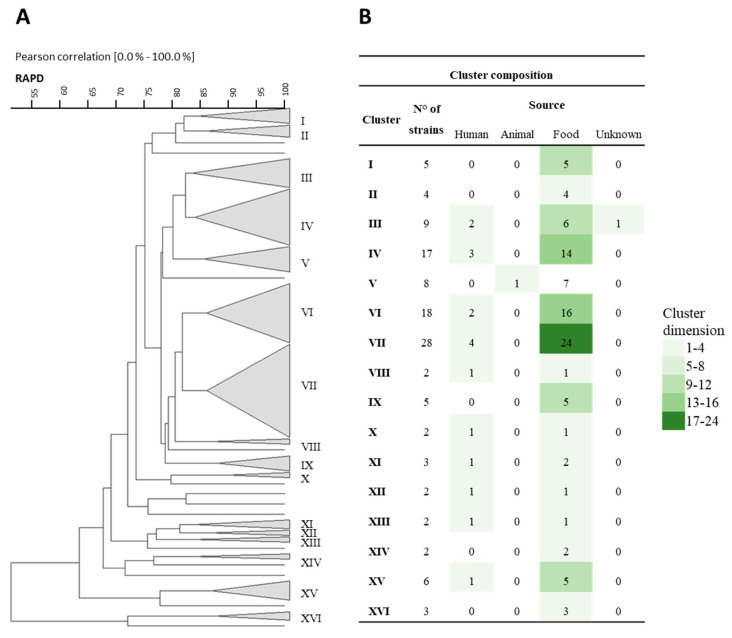
(**A**) Cluster analysis using Pearson product-moment correlation coefficients and unweighted pair group method using an arithmetic average (UPGMA) of the profiles obtained by RAPD-PCR analysis of the different tested strains. A similarity coefficient of 83% was arbitrarily chosen. The identified clusters are indicated with Roman numerals. (**B**) The table within the figure shows the composition of the clusters, also in relation to their source of isolation.

Similarly, the RAPD-PCR fingerprints were clustered with an 83% similarity threshold. The strains formed 16 clusters and 13 single strains. The results revealed three primary clusters (IV, VI, and VII), along with others (I, II, III, IX, and XV) (Figure 2A), which again showed a strong correlation with food origin (Figure 2B). 

**Figure 3:**
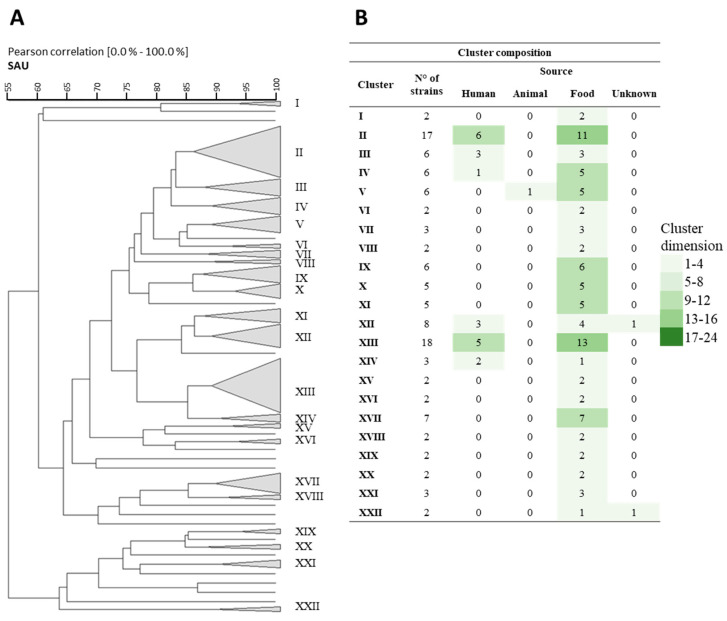
(**A**) Cluster analysis using Pearson product-moment correlation coefficients and unweighted pair group method using an arithmetic average (UPGMA) of the profiles obtained by the SAU-PCR analysis of the different tested strains. A similarity coefficient of 87% was arbitrarily chosen. The identified clusters are indicated with Roman numerals. (**B**) The table within the figure shows the composition of the clusters, also in relation to their source of isolation.

In the Sau-PCR analysis, conducted with a more stringent similarity coefficient of 87%, the comparison of fingerprints resulted in the formation of 22 clusters, leaving 18 strains ungrouped (Figure 3A). Despite the increased discriminatory power of this similarity threshold, it was evident that the primary clusters (II and XIII), as well as others (III and XII), exhibited a heterogeneous composition of strains isolated from both food and human matrices (Figure 3B).

The differences in the obtained results, consistent with findings from the literature utilizing Rep-PCR [29,30,31], RAPD [32], and Sau-PCR [20], testified the high genetic variability of the strains, which was differently detected by the three techniques. Interestingly, no distinct grouping based on species was observed; instead, clustering primarily occurred based on the source of isolation. For instance, the RAPD analysis revealed a similarity of over 80% between the *Lbs. casei*, *Lbs. zeae*, and *Lbs. paracasei* strains isolated from both human faeces and dairy products [33,34], thus resulting in non-discriminatory methodologies. Conversely, a recent study suggests that Rep-PCR and RAPD, in conjunction with other techniques, possess the capability to differentiate between the two species [35].

### 3.2. Stress Resistance

**Figure 4:**
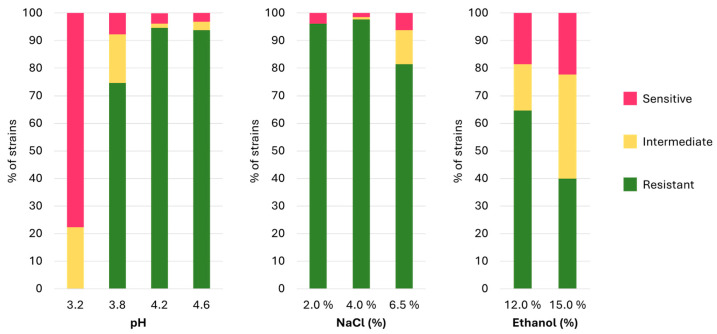
Stress resistance results for pH, NaCl (%), and ethanol (%) expressed as relative percentage of resistant, intermediate, or sensitive strains.

The growth capabilities of various strains may reveal intriguing functional potentials for both probiotic and technological applications. Through the experiments conducted in this study, the capability of the strains to grow at different concentrations of NaCl (2, 4, and 6.5% *w*/*v*), ethanol (12–15% *v*/*v*), and different pH values (3.2, 3.8, 4.2, 4.6) did not show a correlation with their isolation matrix or geographical origin. Instead, the results indicated that resistance to different stresses was a strain-specific trait. In total, only thirteen strains demonstrated resilience to all tested conditions: two *Lbs. casei* (DBPZ0571; N2014) and eleven *Lbs. paracasei* (LMG11961; LMG23518; LMG24101; DBPZ0324; DBPZ0563; DBPZ0579; Q4; M354; I3; DialDan5; DialYak5). As depicted in Figure 4, the majority of the analysed strains exhibited sensitivity to low pH values, results consistent with findings from other studies, in which only few strains managed to thrive at pH values lower than 3.5 [36,37,38,39,40,41], 3.0 [42], or even 2.5 [43], thus confirming the strain-specific nature of these characteristics. 

On the other hand, over 96.5% of the strains demonstrated resistance to NaCl at concentrations of 2% and 4%. However, at a concentration of 6.5%, resistance was observed in 81.5% of the strains. These findings are in agreement with several studies documenting the ability of *Lacticaseibacillus* spp. strains to grow at NaCl concentrations of 6–6.5% [44,45,46], and some strains were also reported to survive above 12% of NaCl [47]. However, other studies have reported either the inability of strains to grow above 4% [48] or, on the contrary, to survive concentrations above 10% [42], emphasizing the strain-specific resistance to this factor, potentially attributable to modifications in cell wall structural properties [49]. Furthermore, 64.6% of the analysed strains exhibited the ability to tolerate ethanol concentrations of 12% *v*/*v*, with 40% extending their tolerance up to 15% *v*/*v* ethanol. These findings are consistent with other studies [50,51], although significant differences in strain responses are evident across the literature for *Lacticaseibacillus* spp.

### 3.3. Antibiotic Resistance 

The importance of studying the resistance profiles of *Lbs. casei, Lbs. zeae,* and *Lbs. paracasei* lies in their extensive use as microbial starters or probiotics [52], as well as their presence in diverse environments where they may coexist with pathogens without barriers [53]. Lactobacilli are generally recognized as safe (GRAS). However, in certain instances, especially in vulnerable individuals, specific strains have been linked to endocarditis or bacteremia. Therefore, it is imperative to thoroughly assess the resistance factors in strains intended for human use. Intrinsic resistance, such as vancomycin resistance in heterofermentative lactobacilli, is not horizontally transferable, whereas resistance genes acquired via plasmids or transposons can be transferred [12]. For this reason, the European Commission proposed that the safety evaluation of microorganisms used in food production could prioritize the detection of transmissible antibiotic resistance markers [54].

Based on the evidence gathered in this study and consistent with findings from other researchers, it was observed that the majority of the strains (>80%) exhibited resistance to aminoglycosides such as kanamycin [23,44,55], streptomycin [23,44,56], and vancomycin [23,55,57,58], with this resistance being considered intrinsic to the species. Additionally, most strains displayed resistance to tobramycin, although documentation of this resistance in the literature is limited [59], as well as resistance to ofloxacin [60]. However, many strains resulted in being sensitive versus all the other tested antibiotics, as confirmed for *Lactobacillus* spp. by other studies [23,52,53,61].

Among the results obtained, eight strains with particular resistance profiles were identified (Table 2). Among these, *Lbs. paracasei* DBPZ0478 exhibited resistance to most of the tested antibiotics, except for clindamycin, quinupristin/dalfopristin, and rifampicin. Some strains isolated from yogurt showed sensitivity to tobramycin but were resistant to erythromycin. *Lbs. paracasei* DialDan8 emerged as the most sensitive strain, demonstrating kanamycin resistance and intermediate resistance to erythromycin and tobramycin. Surprisingly, *Lbs. paracasei* M308 and DialDan8 resulted in being sensitive to vancomycin. This finding contradicts established knowledge, which suggests that some LABs, including *Lbs. casei* and *Lbs. rhamnosus*, are intrinsically vancomycin resistant [62]. However, in a recent study, also *Lbs. paracasei* subsp. *paracasei* strain L1, resulted in being sensitive to vancomycin [63]. 

### 3.4. Antimicrobial Activity and Bacteriocin Production

Antibiotic resistance has escalated in recent decades due to inappropriate antibiotic usage in humans and animals. To address this issue, researchers are exploring alternative substances to treat and prevent bacterial infections. The utilization of bacteriocins could serve as an alternative to antibiotics. Additionally, the well-documented antimicrobial capabilities of lactic acid bacteria (LABs) could be beneficial against foodborne pathogens and in food preservation [64,65,66,67,68]. The production of lactic acid and hydrogen peroxide by LABs can obstruct the growth of pathogens due to their bactericidal effect. The bactericidal/bacteriostatic mechanism of lactic acid likely involves the cytotoxic properties of non-dissociated lactic acid and the insolubility of dissociated lactate, resulting in cytoplasmic acidification and the disruption of proton gradient forces. Moreover, this process may affect the transmembrane pH gradient and reduce the amount of accessible energy for bacteria to grow [69,70]. 

**Figure 5:**
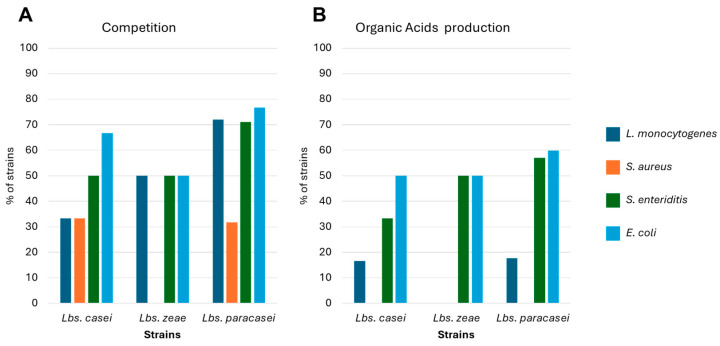
Percentage of *Lbs. casei* and *Lbs. paracasei* strains that showed competition (**A**) against tested pathogens and production of organic acids (**B**).

In this study, the agar well diffusion test was employed to investigate the production of peptidic antimicrobial compounds against *Listeria monocytogenes* ATCC7644, *Staphylococcus aureus* DSM 4910, *Escherichia coli* DSA, and *Salmonella enteritidis* DSA. However, no strains capable of producing bacteriocins have been identified. On the contrary, several strains were capable of directly inhibiting the growth of the four tested pathogens (Figure 5A). Moreover, thanks to the production of organic acids, several strains inhibited the growth of *L. monocytogenes*, *E. coli*, and *S. enteritidis*. However, no inhibition was observed versus *S. aureus* (Figure 5B). These properties have previously been reported in the literature, where in several cases, strains of *Lbs. casei* and *Lbs. paracasei* have demonstrated the ability to inhibit the growth of various pathogens, including *Acinetobacter baumannii*, *Bacillus cereus*, *Campylobacter jejuni*, *Citrobacter freundii*, *Cronobacter sakazakii*, *Enterobacter cloacae*, *Enterococcus faecalis*, *Enterococcus faecium*, *E. coli*, *Klebsiella pneumoniae*, *L. monocytogenes*, *Pasteurella multocida*, *Proteus mirabilis*, *Pseudomonas aeruginosa*, *Salmonella enterica*, *Salmonella typhimurium*, *Staphylococcus aureus*, *Staphylococcus epidermidis*, *Staphylococcus haemolyticus*, and *Streptococcus mutans* [48,58,71,72,73,74]. Furthermore, to contrast the spread of antibiotic resistance, further studies could explore the potential use of these lacticaseibacilli, their supernatants, or cell extracts to counteract the virulence of different pathogens, as reported in several studies [75].

### 3.5. Biogenic Amine (BA) Production 

The decarboxylation of amino acids, particularly in nutrient-scarce environments, can serve as a crucial energy source for microorganisms [76]. Biogenic amines (BAs) are organic bases with aromatic, aliphatic, or heterocyclic structures, commonly found in various beverages and foods. They are primarily generated by the microbial decarboxylation of precursor amino acids [14,77]. Histamine, tyramine, putrescine, and cadaverine are among the BAs typically found in food and beverages. Although their presence often goes undetected due to a lack of associated decline in sensory quality, high consumption of these amines can pose toxicity risks to humans [78,79,80,81,82]. While putrescine and cadaverine are considered non-toxic, they can interfere with histamine and tyramine detoxification processes or potentiate histamine toxicity, respectively. Tyramine ingestion can lead to headaches, vomiting, and hypertension, while histamine can induce breathing difficulties, rash, heart palpitations (allergic-type reactions), low blood pressure, oedema, and vomiting [79,80,83]. The early detection of potential BA-producing bacteria can prevent BA formation and accumulation in food products [78,80] and aid in selecting starter cultures that do not produce BAs [76]. 

Using the method described by Bover-Cid and Holzapfel, 1999, none of the *Lbs. casei* and *Lbs. zeae* strains produced BAs. However, among the *Lbs. paracasei* strains, strain B195 produced tyramine, strain PSG10 produced agmatine, and strain B169 produced both agmatine and tyramine. A multiplex PCR analysis of the genes involved in BA production revealed that all *Lbs. casei* and *Lbs. zeae* strains were negative for all target genes, whereas six strains of *Lbs. paracasei* were positive for the *agdi* gene. Additionally, *Lbs. paracasei* NRRL B-456 tested positive for the *tyrdc* gene and *Lbs. paracasei* B350 was positive for the *hdc* gene. Furthermore, *Lbs. paracasei* PSG10 tested positive for both the *tydc* and *hdc* genes (Table 3). Comparing the results obtained from the two techniques, only one strain yielded concordant results. Despite the identification of these genes through molecular techniques, it was not possible to observe the production of the related BAs. This discrepancy may be attributed to possible gene silencing or very low expression, making it difficult to detect the products using conventional plate growth methods [84,85]. Conversely, according to Buňková et al. (2009), the formation of chemicals with alkaline reactions may have led to colour changes in the pH indicator in the decarboxylase medium, resulting in false-positive reactions [77]. Therefore, PCR analysis could be considered a better and more accurate method for revealing the potential of strains to produce biogenic amines, as it detects strains possessing genes for corresponding enzyme production. However, it should be underlined that the presence of a single gene does not always translate into its expression. Indeed, environmental conditions directly influence microbial metabolism. Therefore, it remains extremely important to test both the genotypic and phenotypic aspects of microorganisms.

### 3.6. arcABC Gene Presence

In this part of the study, the three genes encoding the main enzymes involved in the arginine deiminase (ADI) pathway were investigated: *arcA* (arginine deiminase), *arcB* (ornithine transcarbamylase), and *arcC* (carbamate kinase). During the ADI pathway, lactic acid bacteria produce carbamoyl phosphate and citrulline, which can naturally react with ethanol to form ethyl carbamate, a potential carcinogenic compound found mainly in wine and various fermented foods [86,87]. 

Among all the tested strains for the presence of the *arcA*, *arcB,* and *arcC* genes, only five *Lbs. paracasei* strains (P71; P2P3; B169; B195; DSMZ5622) tested positive for at least one of the genes. Specifically, *Lbs. paracasei* strains P71 and P2P3 (isolated from cheese and goat milk, respectively) were positive for the *arcB* gene, while *Lbs. paracasei* B169, B195, and DSM5622 (isolated from wine, wine, and dairy products, respectively) tested positive for the *arcC* gene. None of the *Lbs. casei* and *Lbs. zeae* strains showed the presence of these genes.

As previously mentioned, one of the main precursors of ethyl carbamate (EC) is citrulline, which could be derived from LAB metabolism. While extensive studies on the presence of these genes in *Lacticaseibacillus* spp. are lacking, some authors have investigated other facultative or obligate heterofermentative LABs. Specifically, *Lentilactobacillus hilgardii*, *Lpb. plantarum*, and *Oenococcus oeni* have been shown to degrade arginine [86,88,89], while *Lentilactobacillus buchneri* has been observed to excrete citrulline [90]. Furthermore, strains belonging to *Levilactobacillus brevis*, *Lentilactobacillus hilgardii*, *Fructilactobacillus florum*, *Llb*. *buchneri*, *Lpb. plantarum*, *O. oeni*, *Pediococcus pentosaceus*, and *Leuconostoc mesenteroides* resulted in being positive to the presence of *arcABC* genes [27,91].

### 3.7. Biogenic Amine (BA)-Degrading Activity: Diaminobenzidine (DAB) Assay and Multicopper Oxidase (MCO) Detection

In fermented foods, particularly those undergoing spontaneous fermentation, the presence of biogenic amines is frequently observed [92]. Individuals with deficiencies in the natural mechanisms of biogenic amine detoxification are particularly vulnerable to these compounds [79,93]. These metabolites not only affect the sensory attributes of the food but also pose a health hazard to consumers, potentially leading to food poisoning [94]. To mitigate these risks, the food industry utilizes specific starter microorganisms carefully selected for their non-production of the amines. These microorganisms not only exert a bioprotective effect by inhibiting the growth of undesired microorganisms but also possess the capability to degrade existing biogenic amines through specialized enzymes [95]. Various methods are employed to control the formation of biogenic amines in food, including the incorporation of additives and preservatives, supplementation with enzymes such as monoamine oxidase (MAO) and diamine oxidase (DAO), and the utilization of bacteria harbouring these enzymes [28,76,93]. MAO and DAO enzymes catalyze the conversion of amines into non-toxic by-products, which can then be safely eliminated from the body [83]. Hence, the ability to degrade biogenic amines was assessed in the tested strains using the DAB assay. While no extracts exhibited bands indicative of MAO/DAO presence, 16 strains displayed mono-copper oxidase (MCO) activity, as detected by DMP staining (Table 4). This characteristic was evidenced by the appearance of yellow-orange bands at consistent positions on the gels, suggesting the presence of similar enzymes across all positive strains. These findings are corroborated by previous studies where several bacterial strains, including *Lbs. paracasei*, which tested negative in the DAB assay, yielded positive results in DMP staining. This underscores the ability of certain *Lacticaseibacillus* spp. strains to utilize histamine, tyramine, putrescine, and DMP as substrates and to catalyze the oxidation of these compounds [28,96]. Given the low number of MCO positive strains, it was not feasible to discern a definitive pattern concerning their origin and isolation source. Nevertheless, six out of the sixteen strains were isolated from dairy products, an ecological niche where strains exhibiting these capabilities have been previously isolated and documented in the literature [97].

### 3.8. Phenotypic Clustering

**Figure 6:**
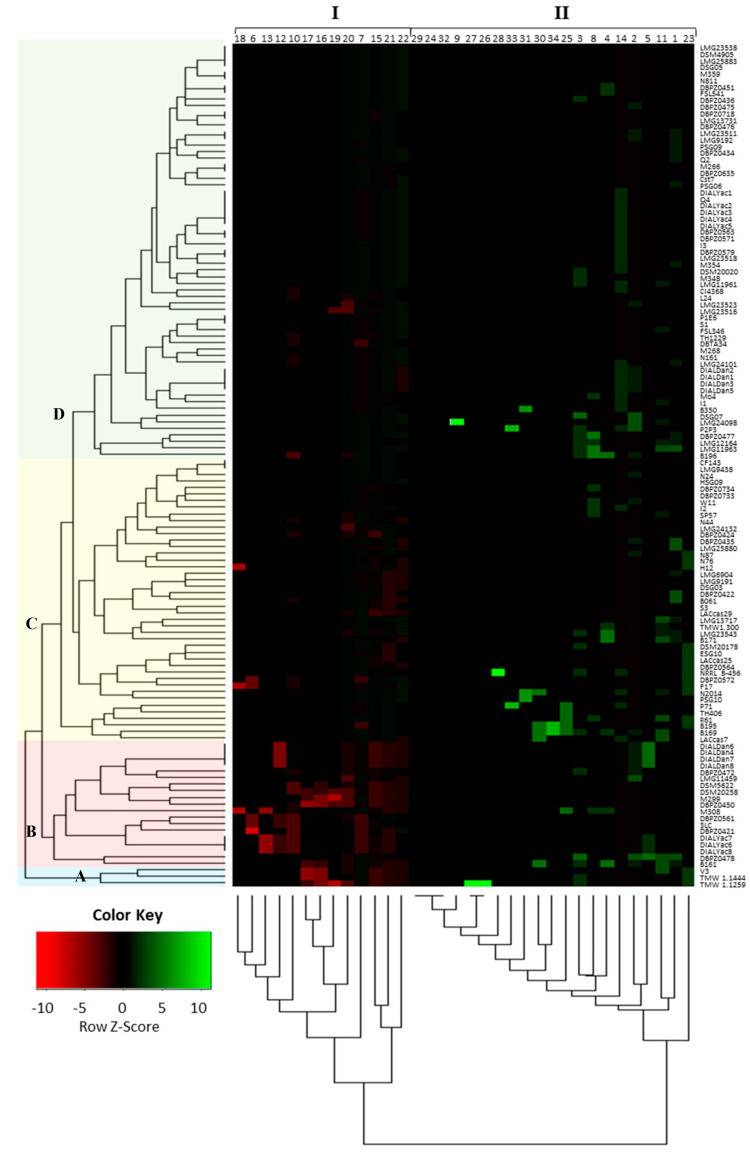
Heatmap of strains obtained by the comparison of the several characteristic studied. Each number indicates a characteristic: 1 to 13, antibiotic resistance (cefoperazone 30 µg, cefazolin 30 µg, chloramphenicol 10 µg, clindamycin 10 µg, erythromycin 30 µg, kanamycin 30 µg, ofloxacin 5 µg, quinupristin/dalfopristin 15 µg, rifampicin 30 µg, streptomycin 25 µg, tetracycline 10 µg, tobramycin 10 µg, and vancomycin 30 µg); 14 to 22, growth abilities (pH 3.2, 3.8, 4.2, and 4.6; NaCl 2%, 4%, and 6.5%; and ethanol 12% and 15%); 23, MCO production; 24 to 27, BA production evaluated using the Bover-Cid et al., 1999, method (histamine, tyramine, putrescine or agmatine, and cadaverine); 28 to 31, genes involved in BA production (the *tyrdc*, *odc*, *agdi*, and *hdc* genes); and 32 to 34, genes involved in ethyl carbamate production (*arcA*, *arcB*, and *arcC* genes). Strain DBPZ0420 did not provide a comparable Rep profile to the other strains, so it was not included in this analysis. The different colours in the left side underline the different clusters A (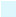), B (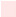), C (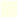), and D (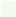).

Based on all the phenotypic characterization results, we conducted a clustering analysis of the different strains. As can be observed in Figure 6, the most discriminating factors were antibiotic resistance and tolerance to various pH, ethanol, and NaCl conditions. The heatmap colour variation reflects the significance of the correlation among these traits. All strains formed interconnected subgroups, but *Lbs. paracasei* TMW 1.1444, TMW 1.1259, and V3 (highlighted in blue) emerged as the most distinct strains across all tested characteristics.

Furthermore, the traits under study segregated into two main clusters (I and II). Cluster I exhibited correlations among almost all growth capabilities (pH tolerance and resistance to different NaCl and ethanol concentrations) along with intrinsic resistance to antibiotics (vancomycin and tobramycin). On the other hand, Cluster II encompassed the presence/absence of genes related to biogenic amines (BAs) and ethyl carbamate production, detection of mono-copper oxidase (MCO), and most antibiotic resistance traits. Additionally, a growth capability at pH 3.2 was found within this second group.

Based on these factors, it was possible to gather the analysed strains in four main clusters: the cluster highlighted in red is made up of strains with low resistance to various stress factors and to some antibiotics. On the contrary, it is possible to see how all the other factors analysed were distributed unevenly within the considered strains, underlining once again the strain-specificity of many of the characteristics analysis. Finally, also in this case, it was not possible to observe a clear distinction between the phenotypic characteristics of *Lbs. casei*, *Lbs. zeae,* and *Lbs. paracasei*, with a mixture of the different species among all the different clusters obtained.

However, given the high genotypic and phenotypic heterogeneity of the strains, these clusters do not consist of strains with completely homogeneous characteristics. In this context, representing all tested characteristics through a heatmap can provide a valuable tool following these screening studies. This can facilitate the selection of the best strain based on the desired traits for each potential technological application.

## 4. Conclusions

In this study, 121 *Lbs. paracasei* strains isolated from various origins or acquired from international collections were genetically and phenotypically characterized to assess their technological and safety properties, comparing them to 6 *Lbs. casei* and 2 *Lbs. zeae* strains. The application of these techniques revealed a significant degree of genetic variability, resulting in numerous clusters from Rep-PCR, RAPD, and Sau-PCR analyses, often associated with the source of isolation but not delineating the different species.

The assessment of safety traits and technological properties uncovered substantial variability in strain behaviours, confirming the strain-specific nature of these attributes. Notably, certain strains exhibited unique characteristics with broad potential applications. For example, *Lbs. casei* N2014 showed the *agdi* and *hdc* genes alongside MCO activity, while *Lbs. paracasei* B169 and B195 harboured the *arcC* and *agdi* genes, with the latter also showcasing MCO activity.

Moreover, the identification of specific strains resistant to a wide spectrum of antibiotics, such as *Lbs. paracasei* DBPZ0478, underscores the importance of understanding microbial resistance profiles.

The extensive genetic diversity among the strains highlights their vast potential applications. With the increasing prominence of functional foods and probiotics, the availability of diverse strains facilitates tailored product development. For instance, formulations targeting individuals with heightened susceptibility could utilize strains selectively sensitive to antibiotics, while strains with non-transferable resistances could augment certain antibiotic therapies to mitigate adverse effects.

Furthermore, exploring the metabolism of these microorganisms could lead to reduced reliance on food additives. A growing body of research attests to the bioprotective efficacy of these microorganisms, paving the way for their potential substitution or supplementation of antimicrobials and antioxidants, thereby directly benefiting consumer health.

To elucidate the distribution of various traits among strains, the results were consolidated into a unified matrix, accentuating the strain-specificity of the tested attributes. This comprehensive approach unveiled the diverse capabilities of individual strains, providing insights for targeted applications across various domains. Such detailed screening is crucial for characterizing microorganisms with potential probiotic, postbiotic, and gut microbiota-modifying effects, as well as anti-inflammatory potential, opening doors to innovative health interventions and functional food development.

## Figures and Tables

**Table 1:** List of strains studied in this work, with their isolation source.

Origin	Given Identification
**Raw and heat-treated milk, yogurt, milking machines**	***Lbs.*** ***paracasei***: LMG9192 ^1^, DSM5622 ^2^, P1E6 ^3^, P2P3 ^3^, Mo4 ^14^, DialYac1 ^14^, DialYac2 ^14^, DialYac3 ^14^, DialYac4 ^14^, DialYac5 ^14^, DialYac6 ^14^, DialYac7 ^14^, DialYac8 ^14^, DialDan1 ^14^, DialDan2 ^14^, DialDan3 ^14^, DialDan4 ^14^, DialDan5 ^14^, DialDan6 ^14^, DialDan7 ^14^, DialDan8 ^14^
	***Lbs.*** ***paracasei* subsp. *tolerans***: LMG9191 ^1^, DSM20258 ^2^
**Green, creamy, seasoned cheeses** (Italian cheeses: Scamorza, Parmigiano Reggiano, Grana Padano, Spressa, Asiago, Montasio, Canestrato di Moliterno, Morlacco, Bellunese, Pecorino, Caciocavallo, Provolone, Emmenthal, Raclette de Savoie; Chinese and Tunisian cheeses)	***Lbs.*** ***zeae***: CI4368 ^11^
	***Lbs.*** ***paracasei***: LMG 6904 ^1^, LMG25880 ^1^, LMG25883 ^1^, LMG12164 ^1^, DBPZ0420 ^7^, DBPZ0421 ^7^, DBPZ0422 ^7^, DBPZ0424 ^7^, DBPZ0434 ^7^, DBPZ0435 ^7^, DBPZ0450 ^7^, DBPZ0451 ^7^, DBPZ0472 ^7^, DBPZ0475 ^7^, DBPZ0476 ^7^, DBPZ0477 ^7^, DBPZ0478 ^7^, DBPZ0635 ^7^, DBPZ0733 ^7^, DBPZ0734 ^7^, M266 ^7^, M268 ^7^, M299 ^7^, M308 ^7^, M348 ^7^, M354 ^7^, M359 ^7^, S1 ^7^, S3 ^7^, V3 ^7^, W11 ^7^, DSG03 ^7^, DSG05 ^7^, DSG07 ^7^, ESG10 ^7^, HSG09 ^7^, PSG06 ^7^, PSG09 ^7^, PSG10 ^7^, P71 ^8^, TH1229 ^8^, SP57 ^8^, L24 ^8^, TH406 ^8^, FSL346 ^9^, FSL451 ^9^, DBPZ0436 ^7^, TMW1.1444 ^5^, TMW1.1259 ^5^, LACcas7 ^6^, Cst7 ^10^, 3LC ^10^, DBPZ0718 ^7^, CF143 ^11^, R61 ^12^, F17 ^12^, N24 ^14^, H12 ^12^
**Fermented sausages**	***Lbs.*** ***paracasei***: CTC1675 ^13^
**Sourdoughs**	***Lbs.*** ***casei***: DBPZ0571 ^7^
	***Lbs.*** ***paracasei***: DBPZ0561 ^7^, DBPZ0572 ^7^, Q2 ^7^, Q4 ^7^, I1 ^4^, I2 ^15^, DBPZ0563 ^7^, DBPZ0564 ^7^, DBPZ0579 ^7^, I3 ^15^
**Wine, must, cellar equipment**	***Lbs.*** ***paracasei***: LMG11961 ^1^, LMG11963 ^1^, LMG13717 ^1^, LMG13731 ^1^, B061 ^16^, B161 ^16^, B169 ^16^, B171 ^16^, B195 ^16^, B196 ^16^, B350 ^16^
**Beer, malt**	***Lbs.*** ***paracasei***: LACcas25 ^6^, LACcas29 ^6^, TMW 1.300 ^5^
**Corn steep liquor**	***Lbs.*** ***zeae***: DSM20178 ^2^
**Humans** (saliva, dental caries, blood, urethra, faeces of infants and adults)	***Lbs.*** ***casei***: LMG23516 ^1^, N87 ^15^, N811 ^15^*,* DSM4905 ^2^*,* N2014 ^15^
	***Lbs.*** ***paracasei***: DSM20020 ^2^, LMG9438 ^1^, LMG11459 ^1^, LMG23511 ^1^, LMG23518 ^1^, LMG23523 ^1^, LMG23538 ^1^, LMG23543 ^1^, LMG24098 ^1^, LMG24101 ^1^, LMG24132 ^1^, DBTA34 ^17^, N161 ^15^, N42 ^15^, N44 ^15^, N76 ^15^*,*
**Unknown**	***Lbs.*** ***paracasei***: NRRL B-456 ^18^

^1^ LMG: BCCM/LMG, Belgian Co-ordinated Collections of Micro-organisms (BCCM™), Ghent, Belgium. ^2^ DSM: DSM, Deutsche Sämmlung von Mikroorganismen und Zellkülturen, Braunschweig, Germany. ^3^ Dipartimento di Agraria, Università degli Studi di Sassari, Sassari, Italy. ^4^ Harmonium International Inc., Mirabel, QC, Canada. ^5^ Lehrstuhl für Technische Mikrobiologie, Technische Universität München, Freising, Germany. ^6^ Dipartimento di Scienze e Tecnologie Alimentari e Microbiologiche, Università degli Studi di Milano, Italy. ^7^ Scuola di Scienze Agrarie, Alimentari e Ambientali, Università degli Studi della Basilicata, Potenza, Italy. ^8^ Università degli Studi di Verona, Dipartimento di Biotecnologie, Strada le Grazie 15, Verona, Italy. ^9^ Istituto Zooprofilattico Sperimentale della Sardegna, Sassari, Italy. ^10^ Istituto sperimentale Lattiero Caseario—I.L.C., Lodi, Italy. ^11^ Dipartimento di Scienze e Tecnologie Agro-Alimentari, Unversità degli Studi di Bologna, Bologna, Italy. ^12^ Dipartimento di Scienze delle Produzioni Agrarie e Agroalimentari, Università degli Studi di Catania, Catania, Italy. ^13^ Institut de Recerca I Technologia Agroalimentaries (IRTA), Lleida, Spain. ^14^ Dipartimento di Scienze degli Alimenti, Università degli studi di Udine, Udine, Italy. ^15^ Dipartimento di Agricoltura, Ambiente e Alimenti, Unversità degli Studi del Molise, Campobasso, Italy. ^16^ Institute for Wine Biotechnology Department of Viticulture and Oenology, Stellenbosh University, South Africa. ^17^ Dipartimento di Biotecnologie, Università degli Studi di Verona, Verona, Italy. ^18^ ARS Culture (NRRL) Collection, United States Department of Agriculture, USA.

**Table 2:** Differences in antibiotic resistance traits using *Lbs. casei* LMG23516 as reference strain.

Strain	Origin	Antibiotics												
		CFP30	KZ30	C10	DA10	E30	K30	OFX5	QD15	RD30	S25	TE10	TOB10	VA30
*Lbs. casei* LMG 23516	Human faeces	S	S	S	S	S	R	R	S	S	R	S	R	R
*Lbs. paracasei* DBPZ0478	Caciocavallo	R	R	R	S	R	R	R	S	S	R	R	R	R
*Lbs. paracasei* M308	Canestrato Moliterno	S	S	S	I	S	R	R	I	S	I	S	R	S
*Lbs. paracasei* DialDan8	Yogurt	S	S	S	S	I	R	S	S	S	S	S	I	S
*Lbs. paracasei* DialYak4	Yogurt	S	S	S	S	R	R	I	S	S	R	S	S	R
*Lbs. paracasei* DialYak5	Yogurt	S	S	S	S	R	R	I	S	S	R	S	S	R
*Lbs. paracasei* DialYak6	Yogurt	S	S	S	S	S	R	I	S	S	R	S	R	R
*Lbs. paracasei* DialYak7	Yogurt	S	S	S	S	R	R	I	S	S	R	S	S	R
*Lbs. paracasei* DialYak8	Yogurt	S	S	S	S	R	R	I	S	S	R	S	S	R

Legend: CFP30, Cefoperazone 30 µg; KZ30, Cefazolin 30 µg; C10, Chloramphenicol 10 µg; DA10, Clindamycin 10 µg; E30, Erythromycin 30 µg; K30, Kanamycin 30 µg; OFX5, Ofloxacin 5 µg; QD15, Quinupristin/Dalfopristin 15 µg; RD30, Rifampicin 30 µg; S25, Streptomycin 25 µg; TE10, Tetracycline 10 µg; TOB10, Tobramycin 10 µg; VA30, Vancomycin 30 µg. S—red colour, sensitive; R—green colour, resistant; I—yellow colour, intermediate resistance.

**Table 3:** Differences between detected genes and positive reaction in decarboxylase medium in *Lbs. paracasei* strains. Legend: +, positive; −, negative.

	Gene				Biogenic Amine			
Strain	*tyrdc*	*odc*	*agdi*	*hdc*	Tym	Agm or Putr	Putr	Cad
NRRL B-456	+	−	−	−	−	−	−	−
B085	−	−	+	−	−	−	−	−
B161	−	−	+	−	−	−	−	−
B169	−	−	+	−	+	+	−	−
B195	−	−	+	−	+	−	−	−
LacCas7	−	−	+	−	−	−	−	−
B350	−	−	−	+	−	−	−	−
PSG10	+	−	−	+	−	+	−	−
N2014	−	−	+	+	−	−	−	−

**Table 4:** Strains positive for multicopper oxidase activity.

Strain	Source	Strain	Source
*Lbs. zeae* DSM20178	Corn steep liquor	*Lbs. paracasei* R61	Pecorino cheese
*Lbs. casei* N87	Body excreta	*Lbs. paracasei* F17	Pecorino cheese
*Lbs. casei* N2014	Body excreta	*Lbs. paracasei* H12	Pecorino cheese
*Lbs. paracasei* NRRL B-456	Unknown	*Lbs. paracasei* N76	Body excreta
*Lbs. paracasei* DBPZ0564	Altamura sourdough	*Lbs. paracasei* B195	Wine
*Lbs. paracasei* DBPZ0572	Altamura sourdough	*Lbs. paracasei* LACcas25	Elixir
*Lbs. paracasei* V3	Canestrato Moliterno	*Lbs. paracasei* TMW 1.1444	Pecorino cheese
*Lbs. paracasei* ESG10	Parmigiano Reggiano	*Lbs. paracasei* TMW 1.1259	Parmesan cheese

## Data Availability

Further details on the data are available on request.
